# Simplifying oral misoprostol protocols for the induction of labour

**DOI:** 10.1111/1471-0528.14657

**Published:** 2017-05-15

**Authors:** AD Weeks, K Navaratnam, Z Alfirevic

**Affiliations:** ^1^ Department of Women's and Children's Health Institute of Translational Medicine University of Liverpool Liverpool UK

Induction of labour is carried out worldwide for a broad range of maternal and fetal indications, so as to improve pregnancy outcomes. Oral misoprostol has been widely discussed as a method of labour induction. It is recommended for this indication by the World Health Organization (WHO), the International Federation of Gynecology and Obstetrics (FIGO), and the Society of Obstetricians and Gynaecologists of Canada (SOGC).^1^, ^2^, ^3^ A systematic review comparing misoprostol with Foley catheter and dinoprostone induction agents suggests that ‘Oral misoprostol for the induction of labour is safer than vaginal misoprostol and has the lowest rate of caesarean section’.^4^ A recently completed UK National Institute of Health Research (NIHR) funded network and cost‐effectiveness analysis included 31 induction regimes evaluated in 611 trials with over 100 000 trial participants. Titrated low‐dose oral misoprostol was identified as likely to be the most cost‐effective method, and also had a favourable safety profile.^5^, ^6^ Sublingual or buccal misoprostol had significantly higher rates of hyperstimulation. This recent evidence is in contrast with the current National Institute for Health and Care Excellence (NICE) guidelines that do not recommend the use of misoprostol, citing that misoprostol is not labelled for labour induction, and that accurate concentrations and reliable drug delivery cannot be guaranteed given that low‐dose formulations are not available.^7^

‘Oral misoprostol’, however, is not a single entity and systematic reviewers have struggled to cope with the wide variation in protocols (Table 1). Published randomised trials have a wide variety of misoprostol doses (20–200 *μ*g) and frequency of administration (1–6 hourly). Some protocols use a single dose for the whole induction period, whereas others escalate the dose until the desired effect is achieved. Some use misoprostol purely for cervical ripening and replace it with an oxytocin infusion once membrane rupture is feasible, whereas others use oral misoprostol continuously until delivery. But the variation doesn't stop there. Until recently there was no commercially produced low‐dose misoprostol tablet, and so clinicians developed their own ways of preparing and administering the intended dose. Some practitioners divided the small and notoriously crumbly 200‐ or 100‐*μ*g tablets into fragments. Others made up 1‐*μ*g/ml solutions by dissolving tablets in tap water. It is only recently that commercially available 25‐*μ*g tablets have become available (Cipla, India; Azanta A/S, Denmark), but these are not yet widely available.

**Table 1:** Published trials of labour induction using low‐dose (<50 *μ*g) oral misoprostol

Initial dose (*μ*g)	Formulation	Dosing interval (hours)	Max. single dose *μ*g (vol.)	Continued in active phase?	References
5	Titrated low‐dose solution	1	20 (20 ml)	Not stated	Dodd 2006a^14^
10	Titrated low‐dose solution	4	20 (20 ml)	No	Majoko 2002^15^
20	Titrated low‐dose solution	1	50 (50 ml)	No	Rouzi 2014^16^
			80 (40 ml)	No	Souza 2013^17^
			40 (40 ml)	No	Thaisomboon 2012^18^
			60 (60 ml)	Yes, only if augmentation required	Cheng 2008^19^
	Fixed low‐dose solution	2	40 (40 ml)	Yes, only if augmentation required	Hofmeyr 2001^20^
			40 (40 ml)	Yes	Dallenbach 2003^21^
			20 (20 ml)	Yes	Dodd 2006^22^
	Fixed low‐dose solution	2	20 (20 ml)	No	Moodley 2003^23^
25	Fixed low‐dose solution	2	25 (25 ml)	No	Aalami‐Harandi 2013^24^
	Tablet	2	50	Yes	Bricker 2008^25^
		3	25	Yes	De 2006^26^
		4	100	No	Henrich 2008^27^
			25	No	How 2001^28^
		72	25	No	Kipikasa 2005^29^
30	Fixed low‐dose solution	1	30 (30 ml)	No	Zvandasara 2008^30^

Is there evidence to suggest that any of these protocols are superior? Subgroup analyses of some important clinical outcomes show a clear dose effect. For example, when comparing oral misoprostol with dinoprostone, the rate of hyperstimulation increases as the initial dose rises from 25 to 200 *μ*g.^4^ It would therefore appear that there are safety benefits of using doses of 20–25 *μ*g, even if they may result in a slower induction process. This is supported by a systematic review of just the studies that used 20–25 *μ*g of oral misoprostol, which found lower caesarean section and lower hyperstimulation rates compared with standard induction methods.^8^ And whereas in previous studies researchers have been forced to either use cut 200‐*μ*g tablets or solution, high‐quality 25‐*μ*g tablets are now available. Findings from a non‐inferiority randomised controlled trial (RCT) of oral misoprostol 50mcg versus Foley catheter for induction of labour showed equivalent safety and effectiveness,^9^ whereas misoprostol tablets (25 *μ*g) has recently been found to be more an effective than Foley catheter when given orally in a large Medical Research Council (MRC) labour induction study.^10^

The use of regimens in which misoprostol is given every 2 hours is supported by pharmacokinetic studies that show that oral misoprostol reaches its peak serum level within 30 minutes, but that its half‐life is only 90 minutes as misoprostol acid is rapidly metabolised by the liver and excreted by the kidneys.^11^ With oral misoprostol sustained uterine activity is achieved in 90 minutes and the duration of action is approximately 2 hours.^11^ The 4–6 hourly dosage regimens have stemmed from an incorrect assumption that the oral pharmacokinetic data is the same as that for vaginal doses.^11^

It remains untested whether the low dose of oral misoprostol will perform better if titrated to clinical response, and whether there are benefits of continuing its use through to the end of labour. Most studies have used oxytocin to continue with the induction process once cervical ripening is complete. There is no question that there are considerable logistic and safety challenges with both approaches, particularly in low‐resource settings.

Oxytocin infusions are notorious for causing hyperstimulation, especially when, as in many parts of the world, they are used without electronic rate controllers. In settings where labour ward staff numbers are very limited, an oxytocin infusion can run unsupervised for many hours without a member of staff checking on its rate or effect on uterine contractions.^12^ In contrast, a titrated oral dose of misoprostol needs to be regularly administered by a trained member of staff, a factor that forces some kind of regular clinical assessment and stops the induction process in the absence of staff. So there may also be organisational and safety benefits to the use of low‐dose oral misoprostol over oxytocin in low‐resource settings. This is supported by a recent randomised trial of oxytocin versus oral misoprostol 20‐*μ*g solution given every 2 hours, which found no difference in major outcomes, but reduced rates of hyperstimulation in the misoprostol group.^13^ This advantage of low‐dose oral misoprostol is in agreement with a network meta‐analysis that we recently conducted assessing induction of labour methods.^5^

What is the way forward? Although off‐label drug use remains essential in pregnancy (for example with betamethasone for fetal lung maturation), clinicians continue to worry about using an off‐label drug when labelled alternatives are available. The development or import of a commercially available 25‐*μ*g tablet licensed for labour induction would therefore be a major advance and would provide a definitive protocol. Until that time we recommend the use of 25‐*μ*g tablets or solution used every 2 hours. It appears to be safe to use it up to the time of birth rather than simply for cervical ripening; however, use of this ‘extended’ protocol should include close observation and the use of acute tocolytics when hyperstimulation is suspected. There are no direct comparisons of the extended protocol with the standard regimen, nor of the stepped increase in misoprostol dose up to 50 *μ*g. However, they appear to be safe and effective in studies in which they have been used. More research is required into ways of reducing adverse outcomes in high‐risk groups (nulliparous women or those with a scarred uterus), potentially using a combination of mechanical and uterotonic methods.

Ideally, formal pharmacokinetic studies would help to clarify the differences between tablets and oral solution, and to establish the optimal frequency of the lowest effective dose. Regrettably, such studies are unlikely to be supported by pharmaceutical companies as misoprostol is too cheap to justify the investment. Also, the association of misoprostol with termination of pregnancy does not help, despite the fact that its uterotonic properties are life‐saving in many low‐resource settings. Whether public funders in high‐resource settings will rise to the challenge remains to be seen. A large study of a combination of titrated low dose oral misoprostol followed by oxytocin in active phase of labour versus titrated oral misoprostol alone given until birth seems an obvious way forwards.

## Disclosure of interests

Full disclosure of interests available to view online as supporting information.

## Contribution to authorship

ZA had the original idea for the article. The first draft was written by ADW, with KN contributing research on the doses used. All authors contributed with further editing and approved the final version.

## Funding

There was no specific funding source for this work.

## Details of ethics approval

None required.

## References

[1] WHO . WHO Recommendations for Induction of Labour. Geneva: World Health Organization, 2011. 23586118

[2] Society of Obstetricians and Gynaecologists of Canada . SOGC Clinical Practice Guideline No 296. Induction of Labour, 2013.

[3] FIGO . [http://www.figo.org/sites/default/files/uploads/project-publications/Miso/Misoprostol_Recommended%20Dosages%202012.pdf] Accessed 7 July 2016.

[4] Alfirevic Z , Aflaifel N , Weeks A . Oral misoprostol for induction of labour. The Cochrane database of systematic reviews. 2014;(6):Cd001338. 24924489 10.1002/14651858.CD001338.pub3 PMC6513439

[5] Alfirevic Z , Keeney E , Dowswell T , Welton NJ , Medley N , Dias S , et al. Methods to induce labour: a systematic review, network meta‐analysis and cost‐effectiveness analysis. BJOG 2016;123:1462–70. 27001034 10.1111/1471-0528.13981 PMC5021158

[6] Alfirevic Z , Keeney E , Dowswell T , Welton NJ , Dias S , Jones LV , et al. Labour induction with prostaglandins: a systematic review and network meta‐analysis. BMJ 2015;350:h217. 25656228 10.1136/bmj.h217 PMC4353287

[7] National Collaborating Centre for Women's and Children's Health . Clinical Guideline: Induction of Labour. [https://www.nice.org.uk/guidance/cg70/evidence/full-guideline-241871149] Accessed 7 July 2016.

[8] Kundodyiwa TW , Alfirevic Z , Weeks AD . Low‐dose oral misoprostol for induction of labor: a systematic review. Obstet Gynecol 2009;113(2 Pt 1):374–83. 19155909 10.1097/AOG.0b013e3181945859

[9] Ten Eikelder E , Oude Rengerink K , Jozwiak M , De Leeuw JW , De Graaf IM , Van Pampus MG , et al. Induction of labour at term with oral misoprostol versus a Foley catheter (PROBAAT‐II): a multicentre randomised controlled non‐inferiority trial. Lancet 2016;387:1619–28. 26850983 10.1016/S0140-6736(16)00084-2

[10] Mundle S , Bracken H , Khedikar D , Mulik J , Faragher B , Easterling T , et al. Induction of labour in hypertensive women in India: a randomised trial comparing the Foley catheter with oral misoprostol. Lancet 2017;In press.

[11] Tang OS , Gemzell‐Danielsson K , Ho PC . Misoprostol: pharmacokinetic profiles, effects on the uterus and side‐effects. Int J Gynaecol Obstet 2007;99(Suppl 2):S160–7. 17963768 10.1016/j.ijgo.2007.09.004

[12] Lovold A , Stanton C , Armbruster D . How to avoid iatrogenic morbidity and mortality while increasing availability of oxytocin and misoprostol for PPH prevention? Int J Gynecol Obstet 2008;103:276–82. 10.1016/j.ijgo.2008.08.009 18954869

[13] Ho M , Cheng SY , Li TC . Titrated oral misoprostol solution compared with intravenous oxytocin for labor augmentation: a randomized controlled trial. Obstet Gynecol 2010;116:612–8. 20733443 10.1097/AOG.0b013e3181ed36cc

[14] Dodd JM , Crowther C , Robinson J , editors. Oral misoprostol versus intravenous oxytocin for induction of labour folllowing artificial or spontaneous rupture of membranes: A randomised controled trial. Perinatal Society of Australia and New Zealand 10th Annual Congress; 2006: PSANZ.

[15] Majoko F , Zwizwai M , Nystrom L , Lindmark G . Vaginal misoprostol for induction of labour: a more effective agent than prostaglandin F2 alpha gel and prostaglandin E2 pessary. Central Afr J Med 2002;48:123–8. 14562597

[16] Rouzi AA , Alsibiani S , Mansouri N , Alsinani N , Darhouse K , Rouzi AA , et al. Randomized clinical trial between hourly titrated oral misoprostol and vaginal dinoprostone for induction of labor. Am J Obstet Gynecol 2014;210:56.e1. 23999422 10.1016/j.ajog.2013.08.033

[17] Souza ASR , Feitosa FEL , Costa AA , Pereira AP , Carvalho AS , Paixão RM , et al. Titrated oral misoprostol solution versus vaginal misoprostol for labor induction. Int J Gynecol Obstet 2013;123:207. 10.1016/j.ijgo.2013.06.028 24112746

[18] Thaisomboon A , Russameecharoen K , Wanitpongpan P , Phattanachindakun B , Changnoi A , Thaisomboon A , et al. Comparison of the efficacy and safety of titrated oral misoprostol and a conventional oral regimen for cervical ripening and labor induction. Int J Gynecol Obstet 2012;116:13. 10.1016/j.ijgo.2011.07.027 21959071

[19] Cheng SY , Ming H , Lee JC . Titrated oral compared with vaginal misoprostol for labor induction: a randomized controlled trial. Obstet Gynecol 2008;111:119. 18165400 10.1097/01.AOG.0000297313.68644.71

[20] Hofmeyr GJ , Alfirevic Z , Matonhodze B , Brocklehurst P , Campbell E , Nikodem VC , et al. Titrated oral misoprostol solution for induction of labour: a multi‐centre, randomised trial. Br J Obstet Gynaecol 2001;108:952. 10.1111/j.1471-0528.2001.00231.x 11563466

[21] Dallenbach P , Boulvain M , Viardot C , Irion O . Oral misoprostol or vaginal dinoprostone for labor induction: a randomized controlled trial. Am J Obstet Gynecol 2003;188:162–7. 12548212 10.1067/mob.2003.108

[22] Dodd JM , Crowther CA , Robinson JS , Dodd JM , Crowther CA , Robinson JS , et al. Oral misoprostol for induction of labour at term: randomised controlled trial. BMJ (British Medical Journal). 2006;332:509. 16455695 10.1136/bmj.38729.513819.63 PMC1388124

[23] Moodley J , Venkatachalam S , Songca P . Misoprostol for cervical ripening at and near term–a comparative study. South African medical journal = Suid‐Afrikaanse tydskrif vir geneeskunde. 2003;93:371–4. 12830602

[24] Aalami‐Harandi R , Karamali M , Moeini A . Induction of labor with titrated oral misoprostol solution versus oxytocin in term pregnancy: randomized controlled trial. Revista brasileira de ginecologia e obstetricia: revista da Federacao Brasileira das Sociedades de Ginecologia e Obstetricia. 2013;35:60–5. 10.1590/s0100-72032013000200004 23412004

[25] Bricker L , Peden H , Tomlinson AJ , Al‐Hussaini TK , Idama T , Candelier C , et al. Titrated low‐dose vaginal and/or oral misoprostol to induce labour for prelabour membrane rupture: a randomised trial. BJOG 2008;115:1503–11. 18752586 10.1111/j.1471-0528.2008.01890.x

[26] De A , Bagga R , Gopalan S , De A , Bagga R , Gopalan S , et al. The routine use of oxytocin after oral misoprostol for labour induction in women with an unfavourable cervix is not of benefit. Aust NZ J Obstet Gynaecol 2006;46:323. 10.1111/j.1479-828X.2006.00600.x 16866794

[27] Henrich W , Dudenhausen JW , Hanel C , Chen FC . Oral misoprostol against vaginal dinoprostone for labor induction at term: a randomized comparison. Z Geburtshilfe Neonatol 2008;212:183–8. 18956276 10.1055/s-2008-1077027

[28] How HY , Leaseburge L , Khoury JC , Siddiqi TA , Spinnato JA , Sibai BM . A comparison of various routes and dosages of misoprostol for cervical ripening and the induction of labor. Am J Obstet Gynecol 2001;185:911. 11641677 10.1067/mob.2001.117358

[29] Kipikasa JH , Adair CD , Williamson J , Breen JM , Medford LK , Sanchez‐Ramos L , et al. Use of misoprostol on an outpatient basis for postdate pregnancy. Int J Gynecol Obstet 2005;88:108. 10.1016/j.ijgo.2004.10.006 15694083

[30] Zvandasara P , Saungweme G , Mlambo J , Chidembo W , Madzivanzira N , Mwanjira C . Induction of labour with titrated oral misoprostol suspension. A comparative study with vaginal misoprostol. Central Afr J Med 2008;54:43–9. 10.4314/cajm.v54i9-12.62626 21644418

